# Obstetrics and Gynaecology as career maze: Perceptions of medical students in Saudi Arabia

**DOI:** 10.15694/mep.2020.000162.1

**Published:** 2020-08-10

**Authors:** Shazia Iqbal, Khalid Akkour, Bushra Bano, Manal Abdullah Ali Alghamadi, Nadiyah Adnan Al-Hajouj, Balqees Sami Khaza'l Aljasim, Manal Khalid Kamal Ali Elhelow, Atheer Mansour Almutairi

**Affiliations:** 1 Alfarabi College of Medicine Riyadh; 2 King Saud University Riyadh; 3 Allama Iqbal Medical college Lahore

**Keywords:** Career in Obstetrics and gynecology, Attracting and distracting factors for obstetrics and gynecology specialty, Enhancing career interest, Model for career motivation for undergraduates, Mentorship program for medical students, Career counseling in Medical education

## Abstract

This article was migrated. The article was marked as recommended.

Background

Over time, there has been a gradual decline in the selection of obstetrics and gynecology (OBGY) as a career choice worldwide, which is of great concern for the medical educators, stakeholders, and policymakers to optimize the demand of this specialty.

Objective

To explore the perceptions of medical students about the attractive and distracting factors to choose obstetrics and gynecology.

Method

The focused group discussion was conducted from September 2019 to December 2019 and thematic analysis was done based on grounded theory.

Results

In the study, a total of 49 students participated out of 75 expected participants, and the response rate was 65%. Few participants who agreed to continue their careers in this field supported their ideas because of female gender acceptance for this specialty in Saudi culture, opportunity to observe procedures, and family pressures. The main reason was the tough experience during OBGY clinical rotations, the clinician’s attitude towards training at the hospital workplace, and work stress.

Conclusion

There is a profound influence on learning experience during the course and clinical training in hospital settings. The positive impact of teaching faculty, successful stories of patients, and teaching techniques supported by simulations can provoke the enthusiasm in the students. A mnemonic “BE SMART” is proposed to apply by medical educators to optimize the need of OBGY specialists in the future. It applies the abilities of true professionals indicated by doctors as beneficent, educators; enforces to augment the use of simulators as teaching modality; practices of meditation, and affectionate the mutual relationships.

## Introduction

In the current era, choosing the medical specialty for medical school graduates has become highly challenging due to emerging competition among medical domains. The rapid advances in biomedical sciences, innovative treatment modalities, and emerging technologies have made the selection of careers very perplexing. Simultaneously, the tremendous advancements in medical education and digitalization of the learning environment have created a competitive atmosphere for young budding doctors with a vast range of choices ( Abdulghani et al., 2013). Therefore, the selection of a specialty as a career has become a very challenging decision under the influence of these factors.

### Obstetrics and gynecology as a career situation in Western countries

Over time, there is a gradual decline in the selection of obstetrics and gynecology (OBGY) as a career choice world widely ( Leigh, Tancredi and Kravitz, 2009; Mehmood et al., 2012). Obvious apprehensions and dissatisfaction have been found among medical students about this field. This situation is of great concern for the medical educators, stakeholders, and policymakers to optimize the demand of this specialty in the health care system. Moreover, the sustainability of this domain must advance in the medical field.

While exploring the reasons for lack of interest in OBGY, there are various factors which influence the selection or rejection of this specialty. A study determined that OBGY was not a striking specialty for medical students because of stress, extensive working hours, and a hectic lifestyle. Similarly, there is evidence, that the negligible percentage of the subgroup who initially choose OBGY as a career, the majority of them planned to quit it because of extreme stress, fear of litigations, and enormous liabilities in this field ( Deutsch et al., 2007).

It is evident that some OBGY trainees tend to leave the residency program after joining due to an unhealthy working environment, morale, and undermining issues. Besides, the authors identified that lack of academic support, extensive paperwork, overwhelming lifestyle, and lack of motivation reasoned the decision to quit OBGY as a career ( Gafson et al., 2017). It has been found that students’ experience during OBGY rotation during the clerkship phase significantly determined a lot for their selection to choose this specialty in the future ( Hammoud et al., 2006). Better the clinical rotation experience, more chances of adopting the specialty as a career.

### Obstetrics and gynecology as a career in the Middle East

Regarding OBGY as a career choice, the prevailing situation in the Middle East is not very different from that of western countries. Very few studied inside the Middle East region dealt with the research about the influencing factors and persuading expulsion for the selection of medical students to choose obstetrics and gynecology. In Sudan, the researchers found that OBGY specialty is not among the first three choices of career ( Alawad et al., 2015). A study at Saudi Arabia found the declining trend for selection of OBGY by interns and highlighted this is an alarming situation that needed research attention to bring the solution to this problem ( Swaid, Elhilu and Mahfouz, 2017).

Regarding the OBGY career, there was a significant relationship found between the genders in Saudi Arabia. It was determined that the OBGY was chosen by the female mostly as compared to males ( Zolaly, Kasim and Mahmoud, 2013). Similar kind of gender variances was detected during the clerkship phase in medical schools at America and authors prompted to fix this gender discrimination timely by counseling to propagate OBGY as a specialty ( Craig et al., 2018; Lambert, Goldacre and Turner, 2006). The authors found that OBGY clinical rotation was not enjoyable for male students because they were not given much chance to practice due to privacy issues; for instance, the male did not get a chance for gynecological examination especially pelvic examination as compared to that of female students.

Such analysis predicts and speculates the policymakers, researchers, and educators to manipulate the situation to probe underlying reasons for supporting the OBGY specialty. There is a significant concern regarding the development of tools to predict OBGY as a career ( McAlister et al., 2007). Moreover, there is a notable research gap to identify the influencing and distracting factors for determining career choices through in-depth analysis through qualitative research in public and private sectors ( Swaid, Elhilu and Mahfouz, 2017).

**Figure 1.**
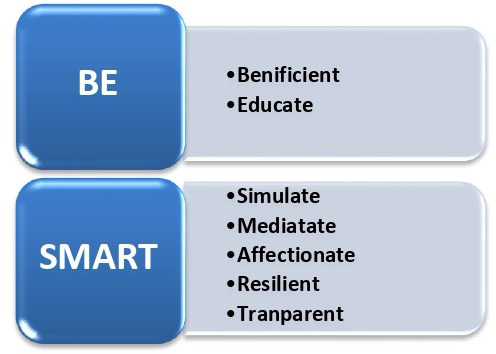
Personal and professional development framework for medical graduates in the form of the mnemonic “BE SMART”

This study explored the insight of medical students in Saudi Arabia about the OBGY field as the choice for a career in the future. It explored the attracting and distracting factors to choose a career of obstetrics and gynecology in the future. The authors narrated a personal and professional development framework in the form of the mnemonic “BE SMART” which will serve as a prompt to establish these qualities in the medical graduate at medical schools ( Figure 1).

The medical graduates ought to “BE SMART”; Beneficent, Educate, Simulate, Meditate, Affectionate, Resilient, and Transparent. This mnemonic spells out all the key qualities for a medical graduate to develop and possess in their personality. Enforcement and establishment of such abilities in the medical graduates during the program can enable them to create interest in OBGY. Moreover, this framework can be applied to other medical and surgical domains apart from the OBGY specialty, where health professions are frequently encountering emergencies and unpredictable outcomes during patients care. We found this model very valuable and applicable during career counseling sessions. Additionally, we proposed a strategic “3D” model to be adopted by medical educators and teaching faculty for the development of medical student’s interest in Obstetrics & Gynecology specialty. Here the “3D” model stands for:

1. Develop engaging learning strategies during clinical placements
2. Display successful stories of patients to encourage students
3. Deploy focused mentorship sessions to enhance career intelligence in students

**Figure 2.**
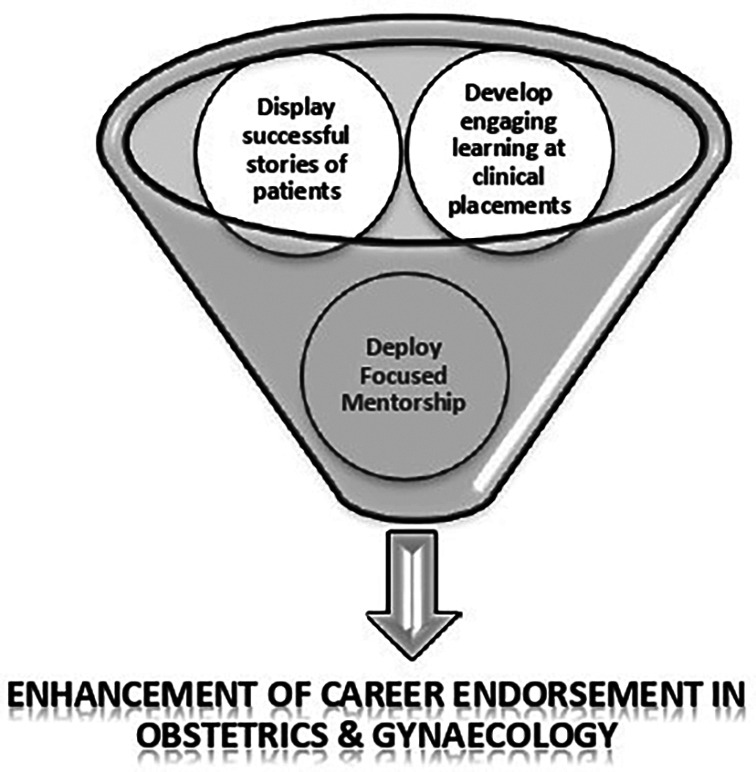
Strategic “3D” Model for medical educators/faculty to promote the medical student’s interest in Obstetrics & Gynecology specialty

## Methods

A qualitative study was done from September 2019 to December 2019, through focused group discussion among the final year medical students, at one of the private colleges of medicine, in Saudi Arabia. The college offers a Bachelor in Medicine and Surgery (MBBS) program of 212 credits, full time delivered over six years. OBGY is taught in the fifth year as a longitudinal course over 18 week’s duration and it is six credits course. The course is delivered through interactive lectures, skill lab simulation sessions, and hospital rotation in the obstetrics and gynecology department.

The study involved six groups of male and female students, including eight to ten students in each group with an average GPA. All the participants involved in the study had completed the OBGY course and they are about to complete the MBBS program. The focused group discussion was coded and themes were identified based on the grounded theory. This approach provided a variety of experiences, stories among the participants, and offered a range of information. Human perceptions could be quite variable, so the interpretation of their views was examined using an interpretive approach ( Rahman, 2017). Ethical approval was taken from IRB Alfarabi College of Medicine and formal consent was taken from all participants before the start of interviews. Participants were ensured to keep their identity anonymous.

## Results/Analysis

In the study, a total of 49 students participated out of 75 expected participants, and the response rate was 65%. Regarding the choice of OBGY as a career, among all the groups of male and female students, very few students claimed confidently that they will pursue their careers in obstetrics and gynecology. Most participants considered OBGY as a low priority.

Concerning the reasons for attracting factors, very few female participants who may think to continue their career in this field supported their ideas because of female gender acceptance for this specialty in Saudi culture and family pressures. Moreover, they mentioned that their interest in this subject impact of a tutor, faculty motivation, the enjoyable experience of the course, the opportunity to observe procedures during the clinical placement at the hospital may be the reason to choose OBGY in the future. For instance one of the students mentioned enthusiastically:

“It was a wonderful experience to observe diagnostic laparoscopic procedure.........I have a much better idea of female pelvic anatomy now....... I even waited more than expected to observe a vaginal delivery and at the gyne clinic we observed how to take pap smear, it was so exciting.

Among male students, only a few students considered OBGY career in the future as they found the latest surgical interventional procedures very interesting. They mentioned that as the surgical trends have been changed and innovative approaches for pelvic surgery is through laparoscopy and robotics. Therefore, they may consider this field otherwise they are not much interested in Obstetrics. Moreover, there were cultural issues and significant apprehension about less employability in the future for being selected for the OBGY position was obvious among male participants. Few of the participants stated:

“The use of technology is very much growing in medicine and surgery.......I want to be trained on robotic surgery ......since my teacher showed us a few videos of hysterectomy through robotics, I am impressed and want to follow it............ Even lecture on management of fibroids through MRI waves is astonishing........even embolization of uterine artery is very striking”.

About the reason for distracting aspects, most of the participants agreed that they were interested in the subject itself but not sure to continue this specialty as a career. The main reason was the tough experience during OBGY clinical rotations, clinician’s attitude towards training at the hospital workplace, and the nature of work at hospital settings. For instance, few of the students expressed despondently:

“It was not a good experience during OBGY rotation because of very long stressful hectic duties, you cannot give much time to the family with such hours. The clinicians remained busy all the time at clinics, wards, and operation theatres. No much time with them for formal teaching at the hospital”.

Such findings instigate the requirement to improve the clinical experience at the hospital. Additionally, participants highlighted that work stress, duty hours, apprehension of malpractice and work-life balance were the significant factors for the disinterest in this field. Their personal experiences of observations in the emergency and labor wards were reflected as distracting factors. In short, it was concluded that besides stress burnout, apprehension of litigations and fear of penalties especially in obstetrics initiated the lack of interest in this field as a career.

## Discussion

On the whole, the situation about OBGY as a career maze needs a comprehensive analysis to strategically anticipate and promote its growth. There has been trepidation for the blooming of OBGY for a long time and researchers kept on thriving to make it an attractive field ( Gibbons, 2003). In this study, we found that medical students are lacking interest in this field which predicts further decline in OBGY specialists in the region ( Alshahrani et al., 2014). Such a situation could be challenging for policymakers and stakeholders to dig more about underlying causes and address them timely.

Our study determined that the most influential predictors for choosing obstetrics and gynecology specialty may be the student’s experience during clinical rotation and as a whole OBGY course experience during MBBS. The healthier the experience better will be the perceptions to opt-in this field. Once students go through obstetrics and gynecology rotations, they develop their ability to reflect on their experience and think critically about the specialty choices. Therefore, clinical placement in OBGY has a great impact on a student’s wisdom to decide about their future career ( Zahid et al., 2015). We prompted to develop engaging, interactive, learning, and teaching methods at clinical placements to make student’s learning experience more enjoyable and exciting ( Figure: 2).

### Gender discrimination & bias

There is a considerable gap in the scientific work about the focus of future research on recognizing the reasons for gender discrimination in OBGY. Although some male students specialize in OBGY despite all the cultural challenges, our study determined that females are more tending to opt OBGY as compared to males because of cultural issues. Cultural beliefs, patient’s perspectives in a conservative culture, and personal values are significant determining factors to select the OBGY career. There must be a revolutionary and innovative shift that focuses on individuals’ career choices to the societal and organizational selection by strategies and awareness with local perspectives. The organizations and employers must be offering equal numbers of posts for both males and females at the time of hiring in the system rather than giving more edge to females for being a female to occupy the slot in OBGY ( Bienstock and Laube, 2005).

Regarding gender bias, the difficulty is not only to get hired in OBGY but also to achieve the leadership position in OBGY is the main concern and needs more exploration to sustain OGBY leadership among health professionals ( Hofler et al., 2016). Besides, a legislative ethos that endorses inflexible and rigid career structures has a much stronger influence over career selection. For example, the organizations that are more tending to support females’ presence in obstetrics rather than males consultants cultivate a very tough situation in the future for OBGY specialty ( Hirayama and Fernando, 2018).

### Stress burnout and apprehension of litigations

For health professionals to maintain healthy and balanced family work life has become extremely difficult due to multitasking, unpredictable circumstances in emergencies, and multiple responsibilities at the workplace. We found that the participants recognized the effects of work-family conflicts may cause an unsatisfied career in OBGY ( Hirayama and Fernando, 2018). Our research determined that stress burnout, apprehension of litigations, and fear of penalties especially in obstetrics instigate one of the major factors for students not to adopt this field as a career ( Bell et al., 2006; Wahlberg, 2018). There is a significant role of meditation and self-awareness especially in the case of post-traumatic stress disorders (PTSD). It is uncommon for OBGY specialists to deal with stressful situations, for instance, maternal or neonatal morbidities/mortalities ( Wahlberg, 2018).

Therefore we suggested for OBGY specialists to train their mind to stay calm in a nerve-racking situation. The authors recommended the practices of meditation to develop resilience to enhance personal and professional development ( Figure 1). Besides, practicing and continuous training on high fidelity simulators can help to reduce the chances of medical errors and negligence. Eventually, the continuous rehearsals will lead to better handling of complex multitasking situations, especially in obstetrics emergencies.

### Mentoring programs & student interest groups

Enhancement of student’s experience through a focused mentorship program during the OBGY clerkship phase may convince them to pursue OBGY as a career. Mentors will serve as motivators and influencers for students to promote interest in the OBGY specialty ( Cain et al., 2001; Gariti, Zollinger and Look, 2005).Hence, we proposed the “3D model” recommended establishing a focused mentorship program and student interest groups to promote self-awareness and motivation during the OBGY career ( Figure 2). Also, the arrangements of obstetrics and gynecology career events, developing the student interest groups symposiums, interactive discussions forums, and arranging motivational talks by keynote speakers, can cultivate the medical student’s attention in this field. Moreover, sharing and presenting success stories of critically ill patients can help to promote the interest in this field. Therefore, just like morbidity and mortality meetings, there should be celebrating days. We proposed to celebrate “CHEER UP” Day prompting (Cherish the Hopes Expectations Eagerness, Resilience, Unpredicted Patient’s stories. This day will be meant to reflect on the good practices and share success stories of unexpected patient cases in OBGY specialty.

### Moral and malpractice avoidance strategy

This study determined that students perceived a marked difference in the OBGY practice in the public and private sectors. The patient physician’s relationship and attitude of physicians at a private clinic are more humble, satisfied, and respectful ( Bell et al., 2006). Moreover, in private sectors, the management approach is very cautious and decisions are more business-minded. For instance, obstetric management for the induction of labor in the previous one lower segment cesarean section tends to differ from that of the public sector by giving a trial of labor by induction. However, private obstetricians tend to defer induction of labor with fear of more complications, litigations and specialists prefer to go for elective cesarean section. Eventually, this apprehensiveness leads to malpractice and low morals values. Therefore, we suggested the framework enforces the application of ethical values: physicians as beneficent, practicing as nonmaleficence, transparent, and affectionate towards patients ( Figure 1).

Assessing the attributes in medical students during selection for the medical profession

Moreover, we suggested that assessing a certain extent of interest in this especially ought to be evaluated at the time of entry in the medical school. The choice of the profession must be inquired at the entry of the program is fundamental, because having a career in the top three priorities can predict the student’s interest in the subject in the future. Assessing these attributes, in the beginning, will help the stakeholders to predict that future strategy for OBGY professionals ( Ismail and Kevelighan, 2020; Ismail and Kevelighan, 2018).

Finally, the responsibility does not lie with the medical schools and training institutions only, but also on the policymakers, stakeholders, and governing bodies (Ministry of Health professionals) ( Scott et al., 2010). The policymakers should plan to train family physicians or general practitioners to build interest in OBGY. As a result of this strategy, the burden of OBGY will be shared at the primary level and community gynecology services can serve effective healthcare. There are success stories that give us a combo flavor to thrive for a career in OBGY and GP ( Gulland, 2018; Magowan, 2005). Promoting this idea will be worthwhile for representatives to assess the personal interest especially at an entry-level in medical school ( Magowan, 2005). Besides, elimination of the gender bias, inequalities, and promoting awareness about the OBGY career should be addressed timely by stakeholders and leadership of health professionals ( Lambert, Smith and Goldacre, 2018; Rippinger et al., 2019).

### Limitations and challenges

The study results reflect one of the private medical schools and the views of a single specialty. We believe that more studies should be done in private and governmental sectors in the region so we can better understand and focus on this alarming issue which will keep the OBGY field secured in the future. Further research is required in other specialties to find the variations in the student’s perceptions in different medical fields. The most important challenge id change in the attitude of faculty towards career counseling and mentoring medical students.

## Conclusion

This study concludes that the attitude of Saudi medical undergraduates towards OBGY specialty as a choice of career is declining and need further research at different medical institutions (public and private) in the kingdom. There is a profound influence on learning experience during the course and training in hospital settings. Moreover, the excellence of teaching faculties/clinicians, bringing successful stories and models to the class, & using innovative techniques supported by simulations will impact the students significantly and will provoke their enthusiasm which will be reflected in a better understanding of OBGY as a specialty. Robust mentorship programs and career counseling are imperative during undergraduate medical education.

Based on the analysis, a mnemonic “BE SMART” is proposed to be applied by medical educators to maintain interest and optimize the need of OBGY specialists in the future. It applies the abilities of true professionals indicated by doctors as Beneficent, Educators; enforces to augment the use of Simulators as teaching modality; to follow the practices of Meditation and Affectionate the mutual relationships and stresses upon developing the unique characteristics as Resilient and Transparent. Moreover, we proposed a strategic “3D” model to be adopted by medical educators and teaching faculty for the development of medical student’s interest in Obstetrics & Gynecology specialty.

## Take Home Messages

• Medical undergraduate’s attitude towards OBGY specialty as a choice of career is declining.
• The learning experience during the OBGY course and training in hospital settings has a profound influence on students to adopt OBGY as a career.
• Motivation by teaching faculties/clinicians and sharing success stories of patients can enhance interest in OBGY.
• Robust mentorship programs and career counseling are imperative during undergraduate medical education.
• Use of simulation models in the skill labs and using innovative techniques can have a positive impact on the attitude of students towards the OBGY.
• “BE SMART” is proposed to be applied by medical educators to maintain interest and optimize the need of OBGY specialists.

## Notes On Contributors

Dr. Shazia Iqbal is working as Assistant Professor Obstetrics & Gynaecology at Alfarabi College of Medicine, Riyadh, Saudi Arabia. She is the author of this article and developed the study concept/design. She assists in the career development of medical students with a special interest in innovative educational technologies in medical education. While teaching undergraduate medical students, she realized that there is an immense need to enhance career intelligence among medical students. This research work is her first step in this direction. https://orcid.org/0000-0003-4890-5864

Dr. Khalid Akkour is an assistant professor and chief Obstetrics and gynaecology department College of Medicine, King Saud University, Riyadh. He is co-author of this manuscript and has a keen interest in career development for undergraduate medical students.

Dr. Bushra Bano is working as an assistant professor at Allama Iqbal Medical College Lahore, Pakistan. She is co-author of this article and assisted with refining the conception and design of the manuscript.

Dr. Manal Abdullah Ali Alghamadi is a final year medical student at Alfarabi college of medicine Riyadh. She is representative of the student support section. She is co-author of this article and her area of interest is strengthening students’ vision in medical education.

Dr. Nadiyah Adnan Al-Hajouj is a final year medical student in Alfarabi College of Medicine, Riyadh. She is involved in literature review and editing this manuscript.

Dr. Balqees Sami Khaza’l Aljasim is a final year medical student at Alfarabi college of medicine Riyadh. She is a co-author of this article and her main contribution is data analysis of the study.

Dr. Manal Khalid Kamal Ali Elhelow is a final year medical student at Alfarabi College of Medicine Riyadh, Saudi Arabia. Her main contribution is assistance during focus group interviews conduction and data collection.

Dr. Atheer Mansour Almutairi is a final year medical student in Alfarabi College of Medicine, Riyadh. She is involved in editing and reviewing this manuscript.
