# Supplementary material for: Obstetrics and Gynaecology as career maze: Perceptions of medical students in Saudi Arabia
Source: MedEdPublish (2016). 2020 Aug 10;9:162. [Version 1] doi: 10.15694/mep.2020.000162.1 (PMC10702686; doi:10.15694/mep.2020.000162.1)
Supplement: Peer review history

# Reviewer report: Reviewer response for version 1

**DOI:** 10.21956/mep.20133.r31079

**Published:** 2020-08-19

**Authors:** Trevor Gibbs

**Affiliations:** 1 AMEE

This review has been migrated. The reviewer awarded 4 stars out of 5 I enjoyed reading this article and learned a lot from the authors' suggestions regarding increasing the motivation in students re OBGYN asa career.I would agree that the research methodology needs some adjustment and improvement, and one would expect the authors to explore how these conclusions might apply to other schools, not only within Saudi Arabia but throughout the region. I do however feel that the paper provides both thought and ideas regarding the issue of career choice and I would recommend it to curriculum planners and health providers in their search of curriculum improvement.

Reviewer Expertise:

NA

No decision status is available

# Reviewer report: Reviewer response for version 1

**DOI:** 10.21956/mep.20133.r31083

**Published:** 2020-08-15

**Authors:** P Ravi Shankar

**Affiliations:** 1 American International Medical University

This review has been migrated. The reviewer awarded 4 stars out of 5 This is an interesting article exploring undergraduate medical students toward obstetrics and gynecology (OBGYN) as a career choice. The authors carried out multiple focus group discussions with students. I do agree with the study findings that male students get lesser opportunities and exposure in the discipline compared to females. In many parts of the world it is taken for granted that a female student will pursue a career in OBGYN. The demands of the specialty, long working hours, the stress and the risk of litigation have all been mentioned. As mentioned, male students may be more interested in gynecology and the technical aspects of the specialty. Male gynecologists do face a lot of discrimination as mentioned by the authors. I think career choice may be a better word in the title. The standard of written English is good but there are problems in a few places. Language and grammar corrections can strengthen the manuscript. Details of the students who participated in the focus groups will also be helpful. More details of the methods employed and of the focus group discussion guide will be useful.

Reviewer Expertise:

NA

No decision status is available

# Reviewer report: Reviewer response for version 1

**DOI:** 10.21956/mep.20133.r31081

**Published:** 2020-08-12

**Authors:** Sateesh Babu Arja

**Affiliations:** 1 Avalon University School of Medicine

This review has been migrated. The reviewer awarded 3 stars out of 5 It is very interesting article exploring the reasons for declining trend in medical graduates choosing ONGYN as a speciality of choice. The literature review is relevant and update. It is good to see that authors compared the context of Saudi Arabia with other contexts like America. It is not right but it is true that male students get less chances or opportunities compared to female students in OBGYN rotations in USA. Research methodology could have been better. What questions were used for the focused group discussions? Is it structured interviews or semi-structured interviews or completely open and un-structured discussions? Thematic analysis-how it was done? What were the different steps involved in data analysis? How did authors were based on grounded theory or what was the basis for determining reaching saturation? Authors can detail all these details when they revise this paper. Thank you.

Reviewer Expertise:

NA

No decision status is available

# Reviewer report: Reviewer response for version 1

**DOI:** 10.21956/mep.20133.r31080

**Published:** 2020-08-11

**Authors:** Shahzad Ahmad

**Affiliations:** 1 Fatima Memorial medical and dental college Lahore Pakistan

This review has been migrated. The reviewer awarded 5 stars out of 5 The article is very interesting and authors have pointed out an important aspect of undergraduate’s future apprehensions. No doubt, the development of career intelligence and focusing on the student's concerns regarding choice of specialty should be the medical educator's concern. The abstract is clear, methods are used appropriately and results are well written. Information gathering is relevant. The propoed the "3D" model is well justified in views of results and can be genralised in other clinical fields especially emergency medicine. I would strongly recommend this article as it is a useful contribution to the medical field for career enhancement amoung medical students. It will guide medical educators to focus on the student's feelings about career choice. Finding will assist clinical teachers to address the key areas of improvements such as clinical training and education during student placements at the hospital. Moreover, recommendations will benefit medical teachers to identify and regulate student's emotions, reduce stress, defuse conflict and guide for the selection of career.

Reviewer Expertise:

NA

No decision status is available

# Reviewer report: Reviewer response for version 1

**DOI:** 10.21956/mep.20133.r31082

**Published:** 2020-08-11

**Authors:** Dujeepa D. Samarasekera

**Affiliations:** 1 National University of Singapore

This review has been migrated. The reviewer awarded 2 stars out of 5 This is an interesting study exploring medical students from a Saudi medical school regarding their perceptions to choose obstetrics and gynaecology as a future career choice. It would be good if the authors share the survey process a bit more. How the following processes were done - candidates were selected, interviews were conducted (FGDs?, Group Interviews using semi-structured questions?), the analysis was done, how the saturation of data achieved, themes developed, the process of validation of results. Furthermore, the study shares descriptive data on what they feel regarding topics under discussion however it would be more value add if the authors could focus on why they feel or perceive what is described. This is more so since the study design is qualitative rather than quantitative. Thank you and good luck in submitting a revised manuscript.

Reviewer Expertise:

NA

No decision status is available
